# Phlebovirus and *Leishmania* detection in sandflies from eastern Thrace and northern Cyprus

**DOI:** 10.1186/s13071-014-0575-6

**Published:** 2014-12-12

**Authors:** Koray Ergunay, Ozge Erisoz Kasap, Serra Orsten, Kerem Oter, Filiz Gunay, Ayse Zeynep Akkutay Yoldar, Ender Dincer, Bulent Alten, Aykut Ozkul

**Affiliations:** Faculty of Medicine, Department of Medical Microbiology, Virology Unit, Hacettepe University, Morphology Building 3rd Floor, 06100 Sihhiye, Ankara Turkey; Faculty of Sciences, Department of Biology, Division of Ecology, Hacettepe University, Ankara, Turkey; Faculty of Veterinary Medicine, Department of Parasitology, Istanbul University, Istanbul, Turkey; Faculty of Veterinary Medicine, Department of Virology, Ankara University, Ankara, Turkey; Advanced Technology Education, Research and Application Center, Mersin University, Mersin, Turkey

**Keywords:** Phlebovirus, *Leishmania*, Toscana virus, Sandfly, Turkey, Cyprus

## Abstract

**Background:**

Phlebotomine sandflies are vectors of several pathogens with significant impact for public health. This study was conducted to investigate and characterize phlebovirus and *Leishmania* infections in vector sandflies collected in the eastern Thrace region in Turkey and Northern Cyprus, where previous data indicate activity of these agents.

**Methods:**

Field sampling of sandflies was performed at 4 locations in Edirne and Tekirdag provinces of eastern Thrace and at 17 locations in Lefkosa, Girne, Magosa and Guzelyurt provinces of northern Cyprus. In sandfly pools, phlebovirus RNA and Leishmania DNA were screened via a generic polymerase chain reaction (PCR) and kinetoplast minicircle PCR, respectively. Selected sandfly specimens unsuitable for pathogen detection were identified to species level. Cytochrome oxidase 1 gene region was used for DNA barcoding of selected specimens and pathogen positive pools. Positive amplicons were cloned and characterized by sequencing.

**Results:**

A total of 2690 sandflies, collected from Eastern Thrace (15.4%) and Northern Cyprus (84.6%) were evaluated. Morphological examination of 780 specimens from Cyprus exhibited *Phlebotomus perfiliewi* sensu lato (72.6%), *Phlebotomus tobbi* (19.7%), *Phlebotomus papatasi* (2.8%), *Laroussius* sp*.* (1.6%) and *Sergentomyia azizi* (1.6%), *Sergentomyia* sp. (0.9%), *Sergentomyia minuta* (0.5%) and *Phleobotomus jacusieli* (0.1%) species. Pathogen screening was performed in 1910 specimens distributed in 195 pools. In eight pools of *P.tobbi* sandflies collected in Cyprus, *Leishmania infantum* DNA was demonstrated. Toscana virus (TOSV) genotype A sequences were identified in two pools of *P. perfiliewi* s.l. and one pool of *P.tobbi* sandflies from Cyprus. Co-infection of TOSV and *Leishmania infantum* was characterized in a *P.tobbi* pool. Sequences belonging to novel phleboviruses are revealed in three *P. perfiliewi* s.l. pools. One sequence, provisionally named Edirne virus, identified in Edirne province in eastern Thrace, demonstrated the highest rate of genomic similarity to Adria and Salehabad viruses. Furthermore, Girne 1 and Girne 2 viruses, identified in Girne province, revealed similarities to TOSV and Sandfly Fever Sicilian virus and related strains, respectively.

**Conclusions:**

Activity of TOSV genotype A strains in Cyprus and co-infection of sandfly vectors with L. infantum was documented for the first time. Novel phlebovirus strains of unknown medical significance was identified in sampling regions.

## Background

Arthropod-borne pathogens are transmitted biologically among vertebrate hosts by hematophagous arthropod vectors such as mosquitoes, ticks and other biting flies, such as sandflies. Phlebotomine sandflies (Diptera: Psychodidae, Phlebotominae) are small, fragile, nocturnally-active insects with weak direct flight capability, naturally feeding on a wide range of hosts [1,2]. While members of the *Lutzomyia* genus are found in the New World, sandfly species classified in the genera *Phlebotomus* and *Sergentomyia* inhabit the Old World [2,3]. Sandflies exhibit an extensive zone of distribution including southern, southeastern and central Europe, Asia, Africa, Australia, as well as central and south America [1,4].

Phlebotomine sandflies are vectors of several bacterial, parasitic and viral pathogens with significant impact for public health. Among the most wide-spread and well-known are leishmaniases, bartonellosis, and sandfly-borne viral infections due to phleboviruses [1,2]. Phleboviruses are enveloped viruses that possess single-stranded RNA genome in three segments which encode viral polymerase and proteins [5]. They are classified as a genus in the Bunyaviridae family, and comprise over 70 viruses that constitute nine established and several tentative species [5]. Exposure of a susceptible individual to certain phlebovirus isolates may result in a febrile disease called sandfly fever, also known as phlebotomus, papatacci or three-day fever, or neuroinvasive diseases [3,6]. In the Old World, sandfly fever can be caused by several phlebovirus strains such as sandfly fever Sicilian virus (SFSV) and sandfly fever Naples Virus (SFNV), sandfly fever Cyprus virus (SFCV) and sandfly fever Turkey virus (SFTV) [3]. Moreover, Toscana virus (TOSV), Granada virus and Adria virus are reported to be associated with phlebovirus-induced febrile conditions as well [7-9]. Although mortality or residual sequelea are rare, sandfly fever continues to be an highly-incapacitating and debilitating disease that can significantly affect the indigenous populations as well as travellers [3,6]. The best-known phleboviral agent of sporadic seasonal meningitis/meningoencephalitis is TOSV [3,7]. Neuroinvasive TOSV infections can also result in severe or fatal central nervous system involvement, peripheral neurological symptoms and sequelae such as paresis, persistent speech disorders and hearing loss [10-13]. Furthermore, other phlebovirus strains like SFSV, SFTV and Chios virus have sporadically been reported to cause neuroinvasive diseases as well [1,14,15]. Overall, phleboviral infections are endemic in regions where the vector sandfly species circulate [3].

Leishmaniasis, caused by the flagellate protozoans of *Leishmania* genus (Kinetoplastida: Trypanosomatidae), represents another sandfly-borne infection posing a significant public health problem [16]. Leishmaniasis, endemic in over 80 countries, may exhibit a wide spectrum of clinical forms in affected individuals, from relatively mild cutaneous and mucocutaneous lesions to life-threatening visceral disease [2,17]. Leishmaniasis transmission to susceptible vertebrates may be zoonotic, with dogs being the primary domestic reservoir hosts or anthroponotic, depending on the involved parasite species and geographical location [2,16]. An epidemiologic association of *Leishmania* parasites and phleboviruses, due to shared sandfly vectors, has also been reported, with currently unknown implications [18].

The objective of the current study is to investigate phlebovirus and *Leishmania* infections and to characterize circulating strains in vector sandflies collected from several locations of eastern Thrace region in Turkey and northern Cyprus, where preliminary information suggest the activity of these sandfly-borne pathogens [19-21].

## Methods

### Study setting and sample collection

**Figure 1:**
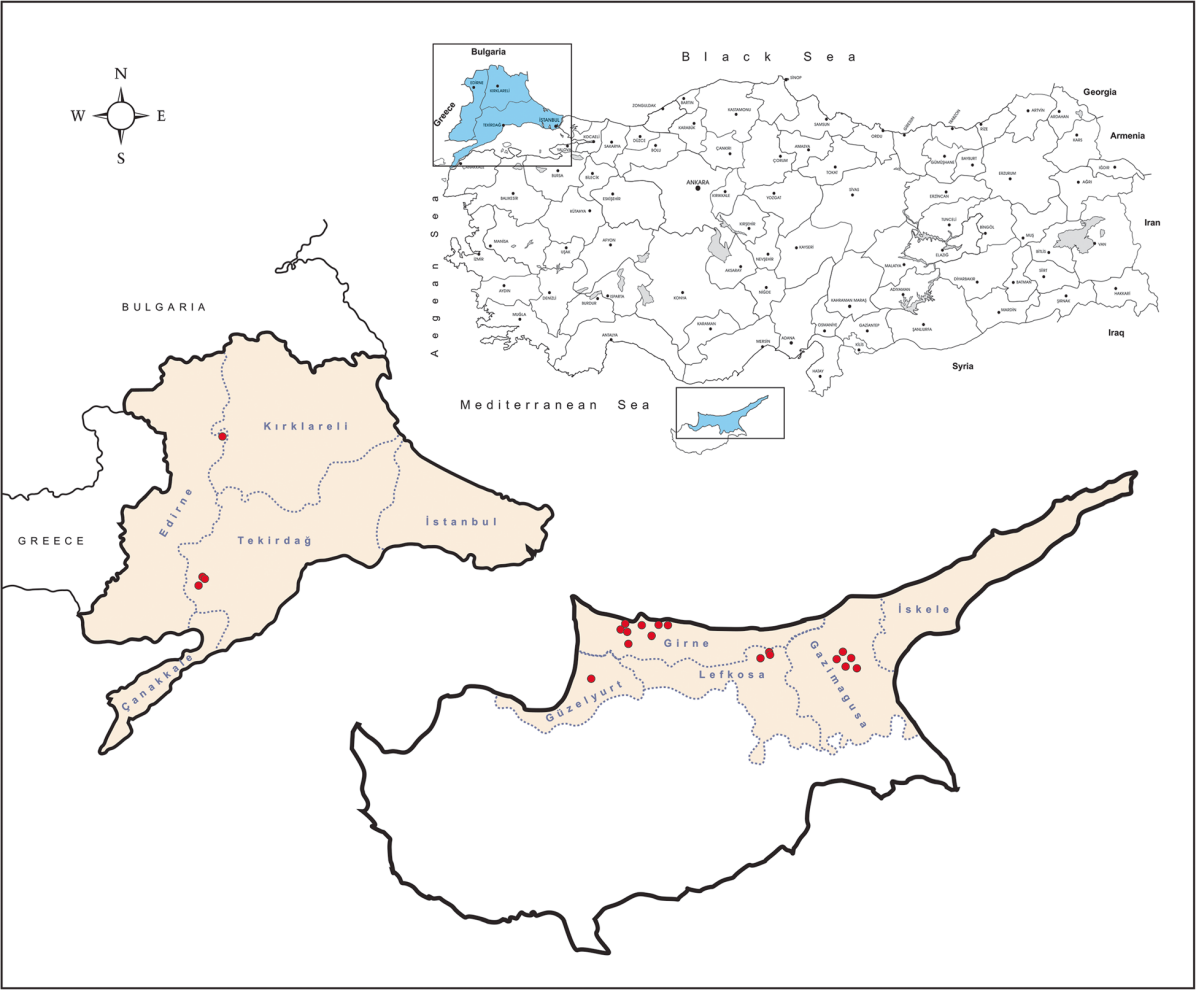
**Illustrative map of locations targeted for sampling in the study.**

The study was undertaken in the eastern Thrace region in Turkey and the Turkish Republic of Northern Cyprus during July, 2013. Eastern Thrace is bordered on the west by Greece and on the northwest by Bulgaria, with the Aegean Sea to the southwest and the Black Sea to the northeast. It has a land area of 23.764 km^2^ (roughly 3% percent of Turkey’s territory) and is separated from Asian Turkey (Anatolia or Asia Minor) by the Sea of Marmara (Figure 1). Edirne and Tekirdag provinces were included in the study from eastern Thrace, whereas in the Turkish Republic of Northern Cyprus, Lefkosa, Girne, Magosa and Guzelyurt provinces covering 76.6% of the land area (2.570/3.355 km^2^) were sampled (Figure 1).

In Turkey, sandfly activity starts in early May and lasts until late October, depending on the region. For a precise sampling strategy, we used the developmental zero value previously calculated for *P. papatasi* [22,23], and estimated that the first adult population of sandflies in the target zones occurs in June to July with a maximum density from July to August and a decrease in September. The field surveys were planned and executed according to these data in order to catch maximum number of sandflies for pathogen detection.

### Sandfly sampling and processing

A total of 21 sites at 6 locations in suburban environments around villages were sampled using CDC Miniature Light Traps, equipped with an ultra-fine mesh (John W. Hock Company, Gainesville, FL) (Table 1). Light traps were placed 1–2 meters above ground in the vicinity or in animal housing facilities in peridomestic sites and left on site from 18:00 to 06:00. Sampling was performed via installing 8 and 17 traps per night in the sampling region in Easthern Thrace and Northern Cyprus, respectively. Captured specimens were collected next morning, kept alive and transferred to the laboratory on ice. Sandflies that were dead upon collection or during transfer were omitted from pathogen detection protocols. These specimens were dissected individually and the head and genitalia were visualized in slides prepared with Swan solution for morphological identification to species level via published keys [24-27]. The remaining body parts were stored in 95% ethyl alcohol for DNA extraction. The specimens, collected and transferred alive, were pooled according to the collection site and date to include 1–25 individuals according to collection date and sex, and stored at −80°C.

**Table 1:** **Sandfly sampling locations and sites employed in the study**

**Location**	**Site**	**Coordinates**	**Altitude (meters)**
Tekirdag province	Izgar1	40°51'39.1840", 26°48'25.6989"	158
	Izgar2	40°51'39.6587", 26°48'18.6922"	211
	Saripolat	40°50'41.2548", 26°46'18.7068"	162
Edirne province	Bostanli	41°36'54.8072", 26°57'58.2783"	100
Lefkosa province	Haspolat	35°13'46.5393", 33°25'07.4721"	139
	Degirmenli1	35°14'40.2355", 33°29'17.3098"	143
	Degirmenli2	35°15'03.7652", 33°29'50.3352"	178
Girne province	Camlibel	35°17'55.3330", 33°02'49.9450"	241
	Gecitkoy1	35°20'21.0144", 33°04'06.1311"	42
	Gecitkoy2	35°20'21.8936", 33°04'01.1799"	51
	Gecitkoy3	35°20'25.3024", 33°04'00.5488"	55
	Karsiyaka	35°21'16.0184", 33°07'45.6885"	23
	Lapta1	35°21'05.3645", 33°08'20.9347"	39
	Lapta2	35°21'06.9917", 33°09'36.2187"	30
	Lapta3	35°21'04.2826", 33°09'47.3865"	31
Magosa province	Gecitkale1	35°16'07.6325", 33°43'18.7998"	85
	Gecitkale2	35°16'07.8482", 33°43'15.2791"	85
	Gecitkale3	35°16'05.7037", 33°43'21.0783"	97
	Gecitkale4	35°16'11.1765", 33°43'12.3796"	90
	Gecitkale5	35°15'48.9168", 33°43'38.1456"	79
Guzelyurt province	Bostanci	35°09'47.2358", 33°01'13.0118"	118

Sandfly pools were homogenized as described previously and clarified by centrifugation at 4000 rpm for 4 minutes [28,29]. Subsequently, each pool was subjected to nucleic acid purification by High Pure Viral Nucleic Acid Kit (Roche Diagnostics, Mannheim, Germany), followed by reverse transcription via random hexamer primers using RevertAid First Strand cDNA Synthesis Kit (Thermo Scientific, Tokyo, Japan).

### Detection of phlebovirus nucleic acids

Consensus degenerate primers targeting the phlebovirus polymerase in the L segment of the viral genome (NPhlebo 1+/1- and 2+/2-) were used in a nested polymerase chain reaction (PCR) in pooled sandflies, as described previously [30]. The expected amplicons of 244 bases were visualized under ultraviolet light after electrophoresis in 2% agarose gels. TOSV ISS.Phl.3 isolate, grown on Vero cells (ATCC CCL81) was used as a positive control and extreme care was taken to prevent carry-over contamination. All amplifications were performed in duplicate.

### Detection of leishmania nucleic acids

The conserved region of the kinetoplast minicircle classes found in all *Leishmania* species was targeted via a previously-described nested-PCR-based schizodeme method, that enables the identification of all *Leishmania* species with clinical impact as well as differentiation of Old World *Leishmania* complexes [31]. The differentiation of Leishmania species is accomplished on the basis of PCR amplicon size, where *L. infantum* generates a 680 bp product whereas 750 and 560 bp products are amplified in *Leishmania tropica* and *Leishmania major*, respectively. The lower detection limit of the assay was reported as 0.1 fg of *L. infantum* DNA [31]. All experiments were performed in duplicate.

### DNA barcoding in sandflies

The cytochrome c oxidase I (COI) gene, widely used for biological barcoding, was targeted in phlebovirus and *Leishmania* positive sandfly pools for species determination as well as selected samples identified morphologically to species level [32]. Dissected thorax and abdomen from individual sandflies, stored in ethyl alcohol, were initially processed with DNeasy Blood & Tissue Kit (Qiagen, Hilden, Germany), prior to amplification. A 658-base pair sequence of the COI gene was amplified with LCO1490 and HCO2198 primers as described previously [32].

### Cloning, sequencing and data analysis

Amplicons obtained from Phlebovirus, *Leishmania* and COI nested PCRs were characterized via sequencing. For this purpose, products were cleaned up using High Pure PCR Product Purification Kit (Roche Diagnostics, Mannheim, Germany), ligated to pJET1.2 vector supplied in CloneJet PCR Cloning Kit (Thermo Scientific, Foster City, CA, USA) and were used to transform cells, as directed by the manufacturers. Forward and reverse primers provided for sequencing were employed for the characterization of cloned amplicons using an ABI Prism 310 Genetic Analyzer (Applied Biosystems, CA, USA). Three to 10 clones were analysed for each target amplicon. Obtained sequences were aligned and analyzed using Bioedit v5.0.5 (http://www.mbio.ncsu.edu/bioedit/bioedit.html), CLC Main Workbench v5.2 (CLCBio, Aarhus, Denmark), and subsequently, by MEGA software v5.2 [33]. Species confirmation of the obtained *Leishmania* sequences was further performed via BLAST (Basic Local Alignment Search Tool) searches in the GenBank (http://blast.ncbi.nlm.nih.gov/Blast.cgi).

## Results

### Sandfly specimens and DNA barcoding

**Figure 2:**
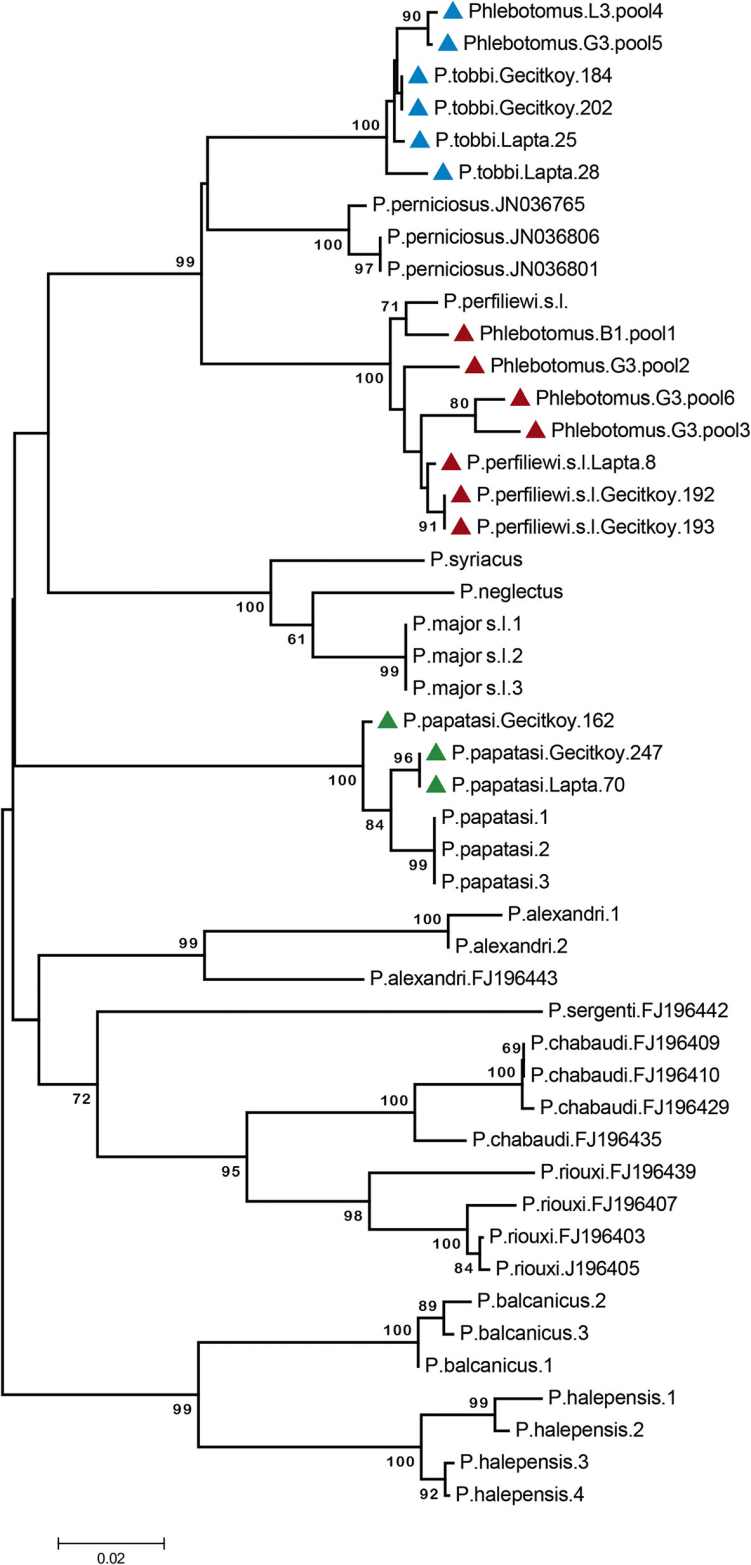
**Neighbour-joining analysis of the partial cytochrome c oxidase I gene sequences obtained from Phlebovirus and** ***Leishmania*** **positive sandfly pools.** Kimura two-parameter distance model was used for taxon identity tree construction. Sequences characterized in this study are marked (coloured triangles) whereas standard sequences are indicated as organism and GenBank accession number (if available).

A total of 2690 sandflies, originating from locations in eastern Thrace (413/2690, 15.4%) and northern Cyprus (2277/2690, 84.6%) were collected in the study. Investigations for Phleboviruses and *Leishmania* were performed in 1910 specimens (1910/2690, 71%), which comprise 413 sandflies (413/1910, 21.6%) collected at locations in eastern Thrace and 1497 (1497/1910, 78.4%) sandflies collected in northern Cyprus (Table 2). Female and male sandflies that comprise 1391 (72.8%) and 519 (27.2%) specimens, respectively, were distributed in a total of 195 pools for pathogen detection. All specimens from eastern Thrace were pooled whereas 780 specimens from northern Cyprus that could not be transferred to the laboratory alive were examined individually for complete morphological identification and DNA barcoding in selected specimens (Table 3). The morphological examination revealed the presence of eight species belonging in the *Phlebotomus* and *Sergentomyia* genera in these specimens (Table 3). The most frequent sandfly species was noted as *P. perfiliewi* sensu lato (72.6%), followed by *P.tobbi* (19.7%), *P. papatasi* (2.8%), *Laroussius* sp*.* (1.6%) and *S. azizi* (1.6%), *Sergentomyia* sp*.* (0.9%), *S. minuta* (0.5%) and *P. jacusieli* (0.1%) (Table 3). DNA barcoding via COI PCR and sequence analysis were performed in 10 randomly-selected sandfly specimens, representing the most frequently-observed species in the sampling locations. These specimens include *P. perfiliewi* s. l. (2 female, 2 male), *P. tobbi* (1 female, 2 male) and *P. papatasi* (1 female, 2 male). Neighbour-joining analysis on the obtained amplicons confirmed the morphological identification (Figure 2).

**Table 2:** **Distribution of sandflies and pools employed for pathogen detection according to sampling sites**

	**Site**	**♀**	**♂**	**Total**	**# of pools**	**Phlebovirus positive**	**Leishmania positive**
Eastern Thrace	Izgar	135	156	291	21	0	0
	Saripolat	1	2	3	2	0	0
	Bostanli	105	14	119	11	1	0
	Total	241	172	413	34	1	0
Northern Cyprus	Degirmenli	24	1	25	3	0	0
	Camlibel	134	35	169	10	0	0
	Gecitkoy	680	190	870	46	4	5
	Lapta	228	77	305	18	1	3
	Gecitkale	54	27	81	13	0	0
	Bostanci	30	17	47	3	0	0
	Total	1150	347	1497	161	5	8

**Table 3:** **Distribution of sandflies with complete morphological identification**

**Species**	**Haspolat**		**Gecitkoy**		**Karsiyaka**		**Lapta**		**Bostanci**		**Total**
	**♀**	**♂**	**♀**	**♂**	**♀**	**♂**	**♀**	**♂**	**♀**	**♂**	
*P. papatasi*	3	2	8	4	-	-	2	-	1	2	22
*P. jacusieli*	-	-	-	1	-	-	-	-	-	-	1
*P. perfiliewi* s.l*.*	-	-	318	218	1	1	1	-	12	15	566
*P. tobbi*	-	-	27	25	3	3	60	35	-	1	154
*Larroussius* sp*.*	-	-	11	-	-	-	2	-	-	-	13
*S. azizi*	-	-	-	-	-	-	7	3	3	-	13
*S. minuta*	-	-	1	-	-	-	1	-	2	-	4
*Sergentomyia* sp*.*	-	-	-	1	-	-	-	5	-	1	7
Total	3	2	365	249	4	4	73	43	18	19	780

### Phlebovirus sequences in sandfly pools

**Figure 3:**
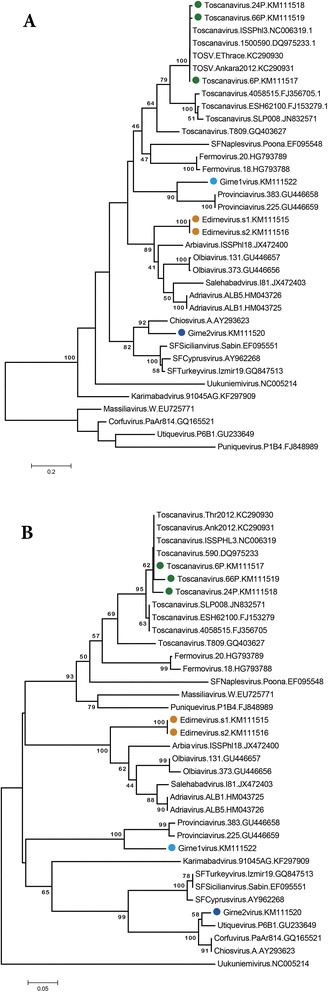
**Neighbour-joining analysis of the Phlebovirus partial nucleotide (A) and aminoacid (B) sequences.** Jukes-Cantor and p-distance models are employed for nucleotide and aminoacid data, respectively. Viruses included in the analysis are indicated with name, isolate identifier and GenBank accession number. Uukuniemi virus is included as an outlier. Sequences characterized in this study are marked (coloured circles). Toscana virus isolates Thr2012 and Ank2012 demonstrate sequences previously characterized in Eastern Thrace and Central Anatolia, Turkey.

Phlebovirus consensus PCR revealed positive results in six pools (6/195, 3.1%) originating from eastern Thrace (1/6) and northern Cyprus (5/6) (Table 2). Sequencing of the cloned amplicons revealed TOSV sequences (GenBank accession numbers: KM111517, KM111518 and KM111519) in three sandfly pools collected in G3 and L3 sampling sites located in Girne province (Table 4). All characterized sequences grouped with TOSV genotype A strains (Figure 3A and B), and demonstrated 0.01% intramural divergence, 97.50-97.92% nucleotide similarity with the prototype strain (TOSV ISS.Phl3) and 99.17% similarity with the genotype A strains previously identified in eastern Thrace and central Anatolia (data not shown). DNA barcoding revealed the infected sandfly species as *P. tobbi* and *P. perfiliewi* s. l. in 2 and 1 pools, respectively (Figure 2, Table 4).

**Table 4:** **Features of sandfly pools positive for phlebovirus RNA**

**Pool no.**	**Sampling site**	**Sampling date**	**Sequence ID**	**Host**	**Pool size**	**DNA barcoding**
1	B1	July 4th, 2013	Edirne virus KM111515, KM111516	*Phlebotomus* spp.	25 (♀)	*P. perfiliewi* s.l.
2	G3	July 11th, 2013	Girne1 virus	*Phlebotomus* spp.	20 (♀)	*P. perfiliewi* s.l.
			KM111522			
3	G3	July 11th, 2013	Girne2 virus	*Phlebotomus* spp.	20 (♀)	*P. perfiliewi* s.l.
			KM111520			
4	L3	July 10th, 2013	Toscana virus	*Phlebotomus* spp.	20 (♀)	*P. tobbi*
			KM111519			
5*	G3	July 11th, 2013	Toscana virus	*Phlebotomus* spp.	20 (♀)	*P. tobbi*
			KM111517			
6	G3	July 11th, 2013	Toscana virus	*Phlebotomus* spp.	20 (♀)	*P. perfiliewi* s.l.
			KM111518			

*positive for *Leishmania infantum*.

One sandfly pool (1/34, 2.9%), collected from the single sampling site in Edirne province, eastern Thrace was positive in the Phlebovirus consensus PCR (Table 2). Sequencing of the cloned amplicons revealed two closely-related sequences (s1 and s2, GenBank accession numbers: KM111515 and KM111516) with 1.6% nucleotide variation resulting in one aminoacid difference (data not shown), which demonstrated limited similarities to previously-described phleboviruses. These novel sequences were observed to have similarities of 74.17-77.08% on the nucleotide and 82.72-85.37% on the aminoacid levels to Adria and Salehabad viruses, respectively (Table 5). Neighbour-joining analyses further supported these sequences to be distinct from other sandfly-borne phleboviruses, with very high bootstrap values and similar tree topologies in various analysis models (Figure 3). These sequences were considered to constitute a novel phlebovirus, tentatively named the Edirne virus, after the sampling province. Amplicons of the DNA barcoding PCR in the Edirne virus-infected sandfly pool were characterized as *P. perfiliewi* s. l. (Figure 2).

**Table 5:** **Pairwise comparison of the partial nucleotide (above diagonal) and aminoacid (below diagonal) sequences of the phleboviruses characterized in the study with various sandfly-borne phleboviruses**

	**Edirne**	**Girne1**	**Girne2**	**TOSV**	**Naples**	**Massilia**	**Punique**	**Fermo**	**Provincia**	**Turkey**	**Cyprus**	**Sicilian**	**Corfu**	**Chios**	**Adria**	**Olbia**	**Salehabad**
Edirne		59.43	56.97	57.38	56.15	32.32	23.53	54.10	35.98	44.44	54.92	55.74	27.76	42.8	74.17	51.46	77.08
Girne1	57.32		60.66	60.66	57.79	34.22	23.51	58.61	46.31	49.18	59.02	59.02	32.70	44.67	53.28	36.89	57.38
Girne2	47.56	54.88		62.08	60.42	33.46	20.00	60.83	33.20	60.66	72.95	70.90	33.84	63.93	49.18	33.61	52.46
TOSV	59.76	63.41	57.32		70.48	35.36	23.53	71.25	39.34	50.82	61.07	61.89	36.88	50.41	54.10	37.30	57.79
Naples	54.88	57.32	54.88	76.83		33.08	21.57	70.42	39.34	48.77	59.02	60.25	35.36	48.77	51.64	36.07	51.23
Massilia	54.88	57.32	58.54	76.83	67.07		45.63	36.12	20.53	27.00	32.32	33.46	66.54	28.80	29.28	19.77	31.18
Punique	41.98	43.90	41.46	63.41	54.88	63.41		20.78	19.91	22.69	23.53	24.31	38.40	21.85	24.39	20.83	19.17
Fermo	56.10	59.76	56.10	85.37	75.61	73.17	57.32		38.93	46.72	57.79	57.79	36.12	47.54	50.82	36.48	54.92
Provincia	32.10	56.10	30.49	34.15	32.93	32.93	40.30	34.15		44.71	37.30	38.11	21.29	40.38	37.66	53.01	37.50
Turkey	51.22	56.10	81.71	59.76	56.10	65.85	41.46	59.76	30.49		81.67	81.25	28.90	75.00	47.23	42.10	45.49
Cyprus	51.22	56.10	82.93	59.76	56.10	65.85	41.46	59.76	30.49	98.78		94.58	32.70	62.50	54.10	37.3	57.79
Sicilian	51.22	56.10	81.71	59.76	56.10	65.85	41.46	59.76	30.49	100.00	98.78		33.46	62.50	54.92	35.25	57.38
Corfu	48.78	54.88	95.12	59.76	56.10	58.54	41.46	57.32	30.49	81.71	82.93	81.71		28.14	25.48	19.39	27.38
Chios	40.00	43.90	82.93	45.12	43.9	46.34	47.89	43.90	35.21	68.29	68.29	68.29	85.37		45.53	39.42	43.03
Adria	82.72	51.22	46.34	54.88	50.00	52.44	41.56	52.44	33.77	50.00	50.00	50.00	46.34	42.86		55.84	78.33
Olbia	56.25	34.15	28.05	36.59	31.71	30.49	38.81	34.15	47.27	28.05	28.05	28.05	28.05	32.39	59.76		53.33
Salehabad	85.37	54.88	51.22	60.98	53.66	56.10	39.02	57.32	31.71	54.88	54.88	54.88	52.44	39.02	97.56	59.76	

GenBank accession numbers of virus sequences included in the table are: Edirne virus: KM111515, Girne1 virus: KM111522, Girne2 virus: KM111520, Toscana virus (isolate ISS.Phl3): NC006319, Sandfly Fever Naples virus (isolate Poona): EF095548, Massilia virus: EU725771, Punique virus: FJ848989, Fermo virus: HG793789, Provincia virus: GU446658, Sandfly Fever Turkey virus: GQ847513, Sandfly Fever Cyprus virus: AY962268, Sandfly Fever Sicilian virus (isolate Sabin): EF095551, Corfu virus: GQ165521, Chios virus: AY293623, Adria virus: HM043726, Olbia virus: GU446657, Salehabad virus: JX472403.

Furthermore, other novel sequences were characterized in two sandfly pools, after the sequencing of the p-hlebovirus amplicons detected in the sampling site G3 in Girne province. The first sequence (GenBank accession: KM111522) demonstrated 59.02% and 60.66% nucleotide similarities to SFSV/SFCV and TOSV, respectively. Moreover, similarities of 59.76% and 63.41% on the aminoacid levels to Fermo virus and TOSV were noted (Table 5). The second sequence (GenBank accession: KM111520) revealed 70.70-72.95% sequence identity to SFSV-SFCV, and 82.93-95.12% aminoacid identity to SFCV and Corfu viruses, respectively (Table 5). These novel sequences were also distinct from each other, having 39.34% divergence on the nucleotide and 45.12% divergence on the aminoacid levels. In the neighbour-joining analyses, the first sequence clusters with but appears distinct from Provincia virus, whereas the second sequence is grouped with Chios or Corfu-Utique viruses, supported again with high bootstrap values (Figure 3A and B). These sequences were tentatively named as Girne 1 and 2 viruses, according to the sampling province. Available homogenates from all phlebovirus positive sandfly pools were inoculated onto Vero cell monolayers, but virus replication could not be detected after several passages. COI barcoding of the sandfly pools with of Girne 1 and 2 virus sequences revealed *P. perfiliewi s*. l., the dominant sandfly species in this sampling location (Figure 2, Table 3).

### *Leishmania* sequences in sandfly pools

Kinetoplast minicircle PCR was positive in 8 pools (8/195, 4.1%) originating from Gecitkoy (5/8) and Lapta districts (3/8) of the Girne province in northern Cyprus (Table 2). Leishmania species in all sandfly pools were characterized as *L. infantum*, via amplicon size and subsequent sequencing. COI barcoding of the positive pools revealed all samples to belong in P. tobbi species. In one of the *L. infantum* positive sandfly pools from Gecitkoy district, TOSV sequences were also characterized, indicating the circulation and probable co-infection of these agents in vectors (Pool no. 5, Table 4).

## Discussion

Recently evidence demonstrates the spread of sandflies as well as sandfly-borne diseases including leishmaniasis and phleboviral infections into previously unaffected regions, such as northern Italy and inland Germany. The ecological expansion of the vectors, mainly driven by environmental factors, usually precedes the emergence of symptomatic infections in human and/or animals [4,17,34]. Entomological surveillance provides crucial information on the circulating pathogens and their vectors, for assessing potential public health threat, for establishing optimal diagnostics and interventions to prevent transmission. The present study was carried out to detect and identify phlebovirus and *Leishmania* strains in two separate regions, the eastern Thrace and northern Cyprus, where reported cases suggest phleboviral infections and leishmaniasis [19,35,36]. It covers a relatively large geographical area including regions with preliminary data as well as previously unexplored locations. To our knowledge, this is the first study undertaken to identify phlebovirus activity and characterize circulating viruses in sandflies in the target regions.

Field sampling of sandflies was performed at four locations in Edirne and Tekirdag provinces in eastern Thrace and at 17 locations in Lefkosa, Girne, Magosa and Guzelyurt provinces in northern Cyprus (Table 1). A total of 2690 sandflies were captured, which comprise 84.6% and 15.4% of the specimens from northern Cyprus and eastern Thrace, respectively. Morphological identification was performed in 780 sandflies (28.9%) collected at various locations in northern Cyprus (Table 3), which were considered as suboptimal for pathogen detection. Among these specimens, sandflies belonging to eight different species were identified, with *P. perfiliewi* sensu lato complex (that comprise *P. perfiliewi perfiliewi*, *P. p. galialeus* and *P. p. transcaucasicus* subspecies) [37] being the most frequent (72.6%), followed by *P. tobbi* (19.7%), *P. papatasi* (2.8%) and others (Table 3). In a previous field survey carried out during 2004 in several districts of northern Cyprus, including the majority of the current sampling locations, all species identified in the present study were reported among 12.517 sandflies collected [38]. Similar to our findings, the presence of *P. perfiliewi* s.l. (as reported as *P. galilaeus*) was recorded and constituted the most abundant species in 80% and 30% of all sampling sites, respectively [38]. Interestingly, 63.8% of all *P. perfiliewi* s.l. specimens were collected from Gecitkoy district, where it provided 56.2% of this species in 2013. Furthermore, *P. tobbi* was recorded in 85% of all sampling sites and it was the most abundant species in Lapta district, which are also comparable to our current findings, where this species were detected in four out of five sampling locations and mainly in Lapta (Table 3). These observations suggest the persistence of similar distribution of sandfly populations in northern Cyprus, despite the relatively low number of sandflies employed for morphological identification and the cross-sectional nature of data collection in this study. Sandfly species inhabiting the southern Cyprus have also investigated in a number of previous studies [39,40]. Here, the presence of eight *Phlebotomus* and three *Sergentomyia* species were reported, and the *Larroussius* species *P. galilaeus* and *P. tobbi* were observed as the most abundant. These data indicate the continuing activity of several sandfly species with vector competence for various pathogens throughout the island. We have further performed COI-based DNA barcoding analysis, which has not been done in previous studies, in a total of 10 individual sandflies that include *P. perfiliewi* s.l., *P. tobbi* and *P. papatasi* specimens, that constitute the frequently-observed species in the sampling locations. This approach provided not only a validation of the morphological identification, but also a basis for identification of the infected pools, as discussed below (Figure 2).

Phlebovirus screening via a nested PCR employing generic primers was performed in 1910 specimens distributed in 195 pools from northern Cyprus (1150 individuals in 161 pools) and eastern Thrace (241 individuals in 34 pools) (Table 2). Phleboviral RNA could be detected in a total of 6 pools (3.1%) that comprise 5 pools (3.1%) from sampling sites in Gecitkoy and Lapta districts of northern Cyprus and 1 pool (2.9%) from Bostanli district of eastern Thrace (Table 2). In 3 (60%) of the infected pools from Cyprus, TOSV partial sequences with very limited intramural nucleotide variations were characterized (Figure 3). This is the first detection of TOSV nucleic acids in the island of Cyprus. Despite the absence of reports involving probable cases in the Turkish Republic of northern Cyprus, virus exposure and seroconversion in a soldier serving in the United Nations forces during 1985 as well as a seroprevalence rate of 20% in a local population were observed in the southern part of the island [41,42]. TOSV is endemic in the Mediterranean region and considered as one of the main central nervous system viral pathogens during sandfly active seasons [1,7]. Genetically-divergent TOSV strains circulate in different endemic regions, with two distinct genotypes or lineages identified [43]. The TOSV genotype A and B strains were mainly isolated in Italy and Spain, respectively, whereas in France, co-circulation of both genotypes have been reported [7,43]. A third genotype, tentatively called as genotype C, is characterized in patients from Croatia [44]. All TOSV sequences identified in sandfly pools in this study belong in genotype A strains, with high rates of similarity to strains of the identical genotype detected previously in Turkey [19,45,46].

TOSV was initially characterized in *Phlebotomus perniciosus* sandflies in Tuscany region of Italy and repeatedly isolated in endemic countries from *P. pernicious* and *P. perfiliewi* s.l., which are considered as vectors [1,3,7,47,48]. Nevertheless, viral genome has also been detected in *Phlebotomus sergenti* and *S. minuta* sandflies, with unknown impact as vectors for human transmission [49,50]. In this study, TOSV-infected pools were characterized as *P. perfiliewi* s.l. in one pool and *P. tobbi* in two pools (Table 5). Although COI barcoding was performed in pools, not in individual specimens, the acquisition of identical sequences from several clones (8–10 for each pool) and the distribution of sandfly species with complete morphological examination support COI findings. *P. tobbi* is a well-known vector of *L. infantum* in the Mediterranean area [51]. In Cyprus, *P. tobbi* is reported as the sandfly vector for canine leishmaniasis, caused by *L. infantum*, and has reviously been isolated from *P. tobbi* in this region [20,39,52]. Although not considered generally as a species with significant anthropophilic behaviour, recent reports indicate that *P. tobbi* can also feed on humans, and is associated with human cutaneous leishmaniasis caused by *L. infantum* in the Cukurova region of Turkey [53]. Moreover, *P. tobbi* is shown to harbor *L. donovani*, an agent of visceral leishmaniasis as well, and is also reported from Cyprus [54,55]. TOSV detection in hematophagous female sandflies in two separate locations implies that *P. tobbi* may also be associated with TOSV transmission. However, data from individual sandflies is required to confirm the probable involvement of *P. tobbi* in TOSV natural transmission cycles.

*Leishmania* screening via a consensus PCR targeting the kinetoplast minicircle in the collected sandflies was also performed in this study. While no detection could be achieved in pools from eastern Thrace, *L. infantum* DNA was characterized in a total of 8 pools collected from Gecitkoy and Lapta districts of Girne province in northern Cyprus (Table 2). All infected pools were further characterized as *P. tobbi* via DNA barcoding. It is well-known that *L. infantum* is mainly responsible for canine leishmaniasis in Cyprus and has been detected in sandflies previously [20,39,52]. Our findings indicate ongoing activity of this agent, observed more frequently than phleboviruses in vectors. Interestingly, concomitant infection of TOSV and *L. infantum* was revealed in a *P. tobbi* pool, collected in Gecitkoy district (Table 4). Transmission of phleboviruses and *Leishmania* parasites via phlebotomine sandflies, sometimes by the identical species, has resulted in the assumption of an epidemiological connection between these agents. Similarities of ecological patterns revealed between Karimabad virus and cutaneous leishmaniasis in Iran provided early evidence [56]. A retrospective serological screening in an endemic region in southern France also indicated a clear relationship between *L. infantum* and TOSV exposure [18]. Moreover, the presence of *P. perniciosus* pools infected either with Massilia virus or *L. infantum* was identified in an urban area in the same region [57]. We have detected co-infections of TOSV and or *L. infantum* in a pool of *P. tobbi* sandflies, demonstrating the activity of these agents in vectors. Since the positive pool consisted of 20 female specimens, it could not be determined whether co-infections originated from a single infected individual or not. Nevertheless, this finding confirms the previous preliminary data suggesting concomitant infections in local sandfly populations likely to transmit both agents. Currently, the impact of these observations is unclear. It needs to be determined whether concomitant or superinfections with these agents result in an increased rate of symptomatic infections or exacerbate clinical symptoms in exposed individuals.

Besides the widely-dispersed SFSV and TOSV, a vast diversity of phleboviruses has been revealed in sandflies in the endemic regions, which include Salehabad, Karimabad and Tehran viruses in Iran, Corfu virus in Greece, Arbia virus in Italy, Massilia virus in France, Granada virus in Spain, Punique and Utique viruses in Tunisia, Adria virus in Albania, as well as other putative isolates [8,28,29,58-64]. Recently-identified Fermo virus in Italy as well as Provencia and Olbia viruses in France and Saddaguia virus in Tunisia have also been included in the list of tentative local viruses [65-67]. Although serologic data indicate human exposure to some of these strains, their pathogenicity and association with clinical disease have not yet been fully elucidated. Some strains are yet to be isolated on cell cultures for complete biochemical and antigenic characterization and lack a full genome sequence [3]. We have detected and characterized three novel phlebovirus sequences in *P. perfiliewi* s.l. pools in this study. The provisionally-named Edirne virus, identified in a location in Edirne province, eastern Thrace, exhibits maximum nucleotide and aminoacid similarities to Adria and Salehabad viruses among other members of the phlebovirus genus (Table 5). Moreover, sequences indicating two distinct strains, named Girne1 and Girne2 viruses were characterized in Gecitkoy district of Girne province of northern Cyprus. Girne 1 virus is phylogenetically-grouped with, but remains distinct from, Provencia virus, identified in P. pernicious sandflies in Provence, southern France [66]. (Figure 3). On the other hand, Girne2 virus groups with Chios virus in nucleotide-based dendrograms and with Chios-Utique-Corfu viruses in aminoacid-based dendrograms, due to the relatively short stretch of the viral genome characterized (Figure 3). SFCV, Corfu, Chios, SFTV, and Utique viruses are closely related to SFSV [3]. SFCV and SFTV are isolated in patients with febrile disease in Cyprus and Turkey, respectively, and Chios virus was characterized as a partial sequence in a patient with severe encephalitis [1,21,68]. Corfu virus was isolated from sandflies belonging to *P. major* s.l. on Corfu Island, and Utique virus was identified as partial sequences in *P. perniciosus* and *Phlebotomus longicuspis* from Tunisia [29,59]. It remains to be elucidated whether Girne1 and Girne2 viruses are capable of, and are responsible for, sandfly fever in Cyprus where previous serological screenings have revealed human exposure to SFSV or antigenically-similar phleboviruses [42].

## Conclusion

TOSV genotype A nucleic acids were detected in *P. perfiliewi* s.l. and *P. tobbi* pools from northern Cyprus, the first characterization of this virus in the region. Ongoing activity *of L. infantum* was observed in this region as well. Co-infections of TOSV and *L. infantum* were demonstrated in a *P. perfiliewi* s.l. pool. TOSV must be considered in the etiology of febrile diseases with/without central nervous system involvement. Three novel phlebovirus strains have been characterized in eastern Thrace and northern Cyprus. The structural aspects and public health impact of these putative strains wait to be investigated fully.

## Abbreviations

COI: Cytochrome c oxidase I
PCR: Polymerase chain reaction
SFCV: Sandfly fever Cyprus virus
SFNV: Sandfly fever Naples Virus
SFSV: Sandfly fever Sicilian virus
SFTV: Sandfly fever Turkey virus
TOSV: Toscana virus

## References

[1] Depaquit J, Grandadam M, Fouque F, Andry PE, Peyrefitte C (2010). Arthropod-borne viruses transmitted by Phlebotomine sandflies in Europe: a review. Euro Surveill.

[2] Maroli M, Feliciangeli MD, Bichaud L, Charrel RN, Gradoni L (2013). Phlebotomine sandflies and the spreading of leishmaniases and other diseases of public health concern. Med Vet Entomol.

[3] Alkan C, Bichaud L, de Lamballerie X, Alten B, Gould EA, Charrel RN (2013). Sandfly-borne phleboviruses of Eurasia and Africa: epidemiology, genetic diversity, geographic range, control measures. Antiviral Res.

[4] Medlock JM, Hansford KM, Van Bortel W, Zeller H, Alten B (2014). A summary of the evidence for the change in European distribution of phlebotomine sand flies (Diptera: Psychodidae) of public health importance. J Vector Ecol.

[5] Plyusnin A, Beaty BJ, Elliott RM, Goldbach R, Kormelink R, Lundkvist A, Schmaljohn CS, Tesh RB, King AMQ, Adams MJ, Carstens EB, Lefkowitz EJ (2012). Bunyaviridae. Ninth Report of the International Committee on Taxonomy of Viruses.

[6] Dionisio D, Esperti F, Vivarelli A, Valassina M (2003). Epidemiological, clinical and laboratory aspects of Sandfly Fever. Curr Opin Infect Dis.

[7] Charrel RN, Bichaud L, de Lamballerie X (2012). Emergence of Toscana virus in the mediterranean area. World J Virol.

[8] Navarro-Mari JM, Gomez-Camarasa C, Perez-Ruiz M, Sanbonmatsu-Gamez S, Pedrosa-Corral I, Jimenez-Valera M (2013). Clinicoepidemiologic study of human infection by Granada Virus, a new Phlebovirus within the Sandfly Fever Naples serocomplex. Am J Trop Med Hyg.

[9] Anagnostou V, Pardalos G, Athanasiou-Metaxa M, Papa A (2011). Novel phlebovirus in febrile child, Greece. Emerg Infect Dis.

[10] Baldelli F, Ciufolini MG, Francisci D, Marchi A, Venturi G, Fiorentini C, Luchetta ML, Bruto L, Pauluzzi S (2004). Unusual presentation of life-threatening Toscana virus meningoencephalitis. Clin Infect Dis.

[11] Martinez-Garcia FA, Moreno-Docon A, Segovia-Hernandez M, Fernandez-Barreiro A (2008). Deafness as a sequela of Toscana virus meningitis. Med Clin (Barc).

[12] Serata D, Rapinesi C, Del Casale A, Simonetti A, Mazzarini L, Ambrosi E, Kotzalidis GD, Fensore C, Girardi P, Tatarelli R (2011). Personality changes after Toscana virus (TOSV) encephalitis in a 49-year-old man: A case report. Int J Neurosci.

[13] Kuhn J, Bewermeyer H, Hartmann-Klosterkoetter U, Emmerich P, Schilling S, Valassina M (2005). Toscana virus causing severe meningoencephalitis in an elderly traveller. J Neurol Neurosurg Psychiatry.

[14] Becker M, Zielen S, Schwartz TF, Linde R, Hofmann D (1997). Pappataci fever. Klin Padiatr.

[15] Ergunay K, Ismayilova V, Colpak IA, Kansu T, Us D (2012). A case of central nervous system infection due to a novel Sandfly Fever Virus (SFV) variant: Sandfly Fever Turkey Virus (SFTV). J Clin Virol.

[16] Lainson R, JJ Shaw JJ, Peters W, Killick-Kendrick R (1987). Evolution, classification and geographical distribution. The leishmaniases in biology and medicine.

[17] Dujardin JC, Campino L, Canavate C, Dedet JP, Gradoni L, Soteriadou K, Mazeris A, Ozbel Y, Boelaert M (2008). Spread of vector-borne diseases and neglect of Leishmaniasis, Europe. Emerg Infect Dis.

[18] Bichaud L, Souris M, Mary C, Ninove L, Thirion L, Piarroux RP, Piarroux R, De Lamballerie X, Charrel RN (2011). Epidemiologic relationship between Toscana virus infection and Leishmania infantum due to common exposure to Phlebotomus perniciosus sandfly vector. PLoS Negl Trop Dis.

[19] Erdem H, Ergunay K, Yilmaz A, Naz H, Akata F, Inan AS, Ulcay A, Gunay F, Ozkul A, Alten B, Turhan V, Oncul O, Gorenek L (2014). Emergence and co-infections of West Nile virus and Toscana virus in Eastern Thrace, Turkey. Clin Microbiol Infect.

[20] Mazeris A, Soteriadou K, Dedet JP, Haralambous C, Tsatsaris A, Moschandreas J, Messaritakis I, Christodoulou V, Papadopoulos B, Ivovic V, Pratlong F, Loucaides F, Antoniou M (2010). Leishmaniases and the Cyprus paradox. Am J Trop Med Hyg.

[21] Papa A, Konstantinou GV, Pavlidou V, Antoniadis A (2006). Sandfly fever virus outbreak in Cyprus. Clin Microbiol Infect.

[22] Erisoz Kasap O, Alten B (2005). Laboratory estimation of degree-day developmental requirements of Phlebotomus papatasi (Diptera: Psychodidae). J Vector Ecol.

[23] Erisoz Kasap O, Alten B (2006). Comparative demography of the sandfly Phlebotomus papatasi (Diptera: Psychodidae) at constant temperatures. J Vector Ecol.

[24] Artemiev MM (1980). A revision of sandflies of the subgenus Adlerius (Diptera, Phlebotominae, Phlebotomus). Zool Zhurnal.

[25] Lewis DJ (1982). A taxonomic review of the genus Phlebotomus (Diptera: Psychodidae). Bull Br Mus Nat Hist (Ent).

[26] Theodor O, Lindner S, Psychodidae-Phlebotominae (1958). Psychodidae-Phlebotominae. Fliegen Der Palearktischen Region.

[27] Killick-Kendrick R, Tang Y, Killick-Kendrick M, Sang DK, Sirdar MK, Ke L, Ashford RW, Schorscher J, Johnson RH (1991). The identification of female sandflies of the subgenus Larroussius by the morphology of the spermathecal ducts. Parassitologia.

[28] Charrel RN, Moureau G, Temmam S, Izri A, Marty P, Parola P, da Rosa AT, Tesh RB, de Lamballerie X (2009). Massilia virus, a novel Phlebovirus (Bunyaviridae) isolated from sandflies in the Mediterranean. Vector Borne Zoonotic Dis.

[29] Zhioua E, Moureau G, Chelbi I, Ninove L, Bichaud L, Derbali M, Champs M, Cherni S, Salez N, Cook S, de Lamballerie X, Charrel RN (2010). Punique virus, a novel phlebovirus, related to sandfly fever Naples virus, isolated from sandflies collected in Tunisia. J Gen Virol.

[30] Sanchez-Seco MP, Echevarria JM, Hernandez L, Estevez D, Navarro-Mari JM, Tenorio A (2003). Detection and identification of Toscana and other phleboviruses by RT-nested-PCR assays with degenerated primers. J Med Virol.

[31] Noyes HA, Reyburn H, Bailey JW, Smith D (1998). A nested-PCR-based schizodeme method for identifying Leishmania kinetoplast minicircle classes directly from clinical samples and its application to the study of the epidemiology of Leishmania tropica in Pakistan. J Clin Microbiol.

[32] Folmer O, Black M, Hoeh W, Lutz R, Vrijenhoek R (1994). DNA primers for amplification of mitochondrial cytochrome c oxidase subunit I from diverse metazoan invertebrates. Mol Mar Biol Biotechnol.

[33] Tamura K, Peterson D, Peterson N, Stecher G, Nei M, Kumar S (2011). MEGA5: Molecular evolutionary genetics analysis using maximum likelihood, evolutionary distance and maximum parsimony methods. Mol Biol Evol.

[34] Maroli M, Rossi L, Baldelli R, Capelli G, Ferroglio E, Genchi C, Gramiccia M, Mortarino M, Pietrobelli M, Gradoni L (2008). The northward spread of leishmaniasis in Italy: evidence from retrospective and ongoing studies on the canine reservoir and phlebotomine vectors. Trop Med Int Health.

[35] Ergin C, Yilmaz S (2002). The regional and seasonal distribution of patients prediagnosed as sandfly fever in Kyrenia, Northern Cyprus. Turk Mikrobiyol Cem Derg.

[36] Töz SO, Ertabaklar H, Göçmen B, Demir S, Karakuş M, Arserim SK, Balcıoğlu IC, Canakçı T, Ozbel Y (2013). An epidemiological study on canine leishmaniasis (CanL) and sand flies in Northern Cyprus. Turkiye Parazitol Derg.

[37] Depaquit J, Bounamous A, Akhoundi M, Augot D, Sauvage F, Dvorak V, Chaibullinova A, Pesson B, Volf P, Léger N (2013). A taxonomic study of Phlebotomus (Larroussius) perfiliewi s. l. Infect Genet Evol.

[38] Demir S, Gocmen B, Ozbel Y (2010). Faunistic study of sand flies in Northern Cyprus. North-West J Zool.

[39] Léger N, Depaquit J, Ferté H, Rioux JA, Gantier JC, Gramiccia M, Ludovisi A, Michaelides A, Christophi N, Economides P (2000). Phlebotomine sandflies (Diptera-Psychodidae) of the isle of Cyprus. II-Isolation and typing of Leishmania (Leishmania infantum Nicolle, 1908 (zymodeme MON 1) from Phlebotomus (Larroussius) tobbi Adler and Theodor, 1930. Parasite.

[40] Depaquit J, Léger N, Ferté H, Rioux JA, Gantier JC, Michaelides A (2001). Economides P:Phlebotomines of the Isle of Cyprus. III. Species inventory. Parasite.

[41] Eitrem R, Vene S, Niklasson B (1990). Incidence of sand fly fever among Swedish United Nations soldiers on Cyprus during 1985. Am J Trop Med Hyg.

[42] Eitrem R, Stylianou M, Niklasson B (1991). High prevalence rates of antibody to three sandfly fever viruses (Sicilian, Naples and Toscana) among Cypriots. Epidemiol Infect.

[43] Collao X, Palacios G, Sanbonmatsu-Gamez S, Perez-Ruiz M, Negredo AI, Navarro-Mari JM, Grandadam M, Aransay AM, Lipkin WI, Tenorio A, Sanchez-Seco MP (2009). Genetic diversity of Toscana virus. Emerg Infect Dis.

[44] Punda-Polic V, Mohar B, Duh D, Bradaric N, Korva M, Fajs L, Saksida A, Avsic-Zupanc T (2012). Evidence of an autochthonous Toscana virus strain in Croatia. J Clin Virol.

[45] Ergunay K, Saygan MB, Aydogan S, Lo MM, Weidmann M, Dilcher M, Sener B, Hascelik G, Pinar A, Us D (2011). Sandfly fever virus activity in Central/Northern Anatolia, Turkey: First report of Toscana virus Infections. Clin Microbiol Infect.

[46] Ocal M, Orsten S, Inkaya AC, Yetim E, Acar NP, Alp S, Erisoz Kasap O, Gunay F, Arsava EM, Alten B, Ozkul A, Us D, Niedrig M, Ergunay K (2014). Ongoing activity of Toscana virus genotype A and West Nile virus lineage 1 strains in Turkey: A clinical and field survey. Zoonoses and Public Health.

[47] Verani P, Ciufolini MG, Nicoletti L, Balducci M, Sabatinelli G, Coluzzi M, Paci P, Amaducci L (1982). Ecological and epidemiological studies of Toscana virus, an arbovirus isolated from Phlebotomus. Ann Ist Super Sanita.

[48] Bichaud L, Dachraoui K, Piorkowski G, Chelbi I, Moureau G, Cherni S, De Lamballerie X, Sakhria S, Charrel RN, Zhioua E (2013). Toscana virus isolated from sandflies, Tunisia. Emerg ınfect Dis.

[49] Es-Sette N, Ajaoud M, Bichaud L, Hamdi S, Mellouki F, Charrel RN, Lemrani M (2014). Phlebotomus sergenti a common vector of Leishmania tropica and Toscana virus in Morocco. J Vector Borne Dis.

[50] Charrel RN, Izri A, Temmam S, de Lamballerie X, Parola P (2006). Toscana virus RNA in Sergentomyia minuta files. Emerg Infect Dis.

[51] Killick-Kendrick R (1990). Phlebotomine vectors of the leishmaniases: a review. Med Vet Entomol.

[52] Deplazes P, Grimm F, Papaprodromou M, Cavaliero T, Gramiccia M, Christofi G, Christofi N, Economides P, Eckert J (1998). Canine leishmaniosis in Cyprus due to Leishmania infantum MON 1. Acta Trop.

[53] Svobodova M, Zidkova L, Dvorak V, Hlavackova J, Myskova J, Seblova V, Kasap OE, Belen A, Votypka J, Volf P (2009). Cutaneous leishmaniasis caused by Leishmania infantum transmitted by Phlebotomus tobbi. Int J Parasitol.

[54] Rioux JA, Leger N, Haddad N, Gramicca M, Jalouk L, Dereure J, Al-Khiami A, Desjeux P (1998). Natural infestation of Phlebotomus tobbi (Diptera, Psychodidae) by Leishmania donovani s. st. (Kinetoplastida, Trypanosomatidae) in Syria. Parassitologia.

[55] Antoniou M, Haralambous C, Mazeris A, Pratlong F, Dedet JP, Soteriadou K (2009). Leishmania donovani leishmaniasis in Cyprus. Lancet Infect Dis.

[56] Saidi S, Tesh R, Javadian E, Sahabi Z, Nadim A (1977). Studies on the epidemiology of sandfly fever in Iran. II. The prevalence of human and animal infection with five phlebotomus fever virus serotypes in Isfahan province. Am J Trop Med Hyg.

[57] Faucher B, Bichaud L, Charrel R, Mary C, Izri A, de Lamballerie X, Piarroux R (2014). Presence of sandflies infected with Leishmania infantum and Massilia virus in Marseille urban area. Clin Microbiol Infect.

[58] Tesh R, Saidi S, Javadian E, Nadim A (1977). Studies on the epidemiology of sandfly fever in Iran. I. Virus isolates obtained from Phlebotomus. Am J Trop Med Hyg.

[59] Rodhain F, Maduloleblond G, Hannoun C, Tesh RB (1985). Corfou Virus - a new phlebovirus isolated from phlebotomine sandflies in Greece. Ann Inst Pasteur Virol.

[60] Collao X, Palacios G, de Ory F, Sanbonmatsu S, Perez-Ruiz M, Navarro JM, Molina R, Hutchison SK, Lipkin WI, Tenorio A, Sanchez-Seco MP (2010). Granada virus: a natural phlebovirus reassortant of the sandfly fever Naples serocomplex with low prevalence in humans. Am J Trop Med Hyg.

[61] Papa A, Velo E, Bino S (2011). A novel phlebovirus in Albanian sandflies. Clin Microbiol Infect.

[62] Moureau G, Bichaud L, Salez N, Ninove L, Hamrioui B, Belazzoug S, de Lamballerie X, Izri A, Charrel RN (2010). Molecular and serological evidence for the presence of novel phleboviruses in sandflies from northerrn Algeria. Open Virol J.

[63] Verani P, Ciufolini MG, Caciolli S, Renzi A, Nicoletti L, Sabatinelli G, Bartolozzi D, Volpi G, Amaducci L, Coluzzi M, Paci P, Balducci M (1988). Ecology of viruses isolated from sand flies in Italy and characterized of a new phlebovirus (Arabia virus). Am J Trop Med Hyg.

[64] Sakhria S, Alwassouf S, Fares W, Bichaud L, Dachraoui K, Alkan C, Zoghlami Z, de Lamballerie X, Charrel RN (2014). Presence of sandfly-borne phleboviruses of two antigenic complexes (Sandfly fever Naples virus and Sandfly fever Sicilian virus) in two different bio-geographical regions of Tunisia demonstrated by a microneutralisation-based seroprevalence study in dogs. Parasit Vectors.

[65] Remoli ME, Fortuna C, Marchi A, Bucci P, Argentini C, Bongiorno G, Maroli M, Gradoni L, Gramiccia M, Ciufolini MG (2014). Viral isolates of a novel putative phlebovirus in the Marche Region of Italy. Am J Trop Med Hyg.

[66] Peyrefitte CN, Grandadam M, Bessaud M, Andry PE, Fouque F, Caro V, Diancourt L, Schuffenecker I, Pagès F, Tolou H, Zeller H, Depaquit J (2013). Diversity of Phlebotomus perniciosus in Provence, southeastern France: Detection of two putative new phlebovirus sequences. Vector Borne Zoonotic Dis.

[67] Fares W, Charrel RN, Dachraoui K, Bichaud L, Barhoumi W, Derbali M, Cherni S, Chelbi I, de Lamballerie X, Zhioua E (2015). Infection os sand flies collected from different bio-geographical areas of Tunisia with phleboviruses. Acta Trop.

[68] Carhan A, Uyar Y, Ozkaya E, Ertek M, Dobler G, Dilcher M, Wang Y, Spiegel M, Hufert F, Weidmann M (2010). Characterization of a sandfly fever Sicilian virus isolated during a sandfly fever epidemic in Turkey. J Clin Virol.

